# The Use of Ultrasound-Based Elastography Techniques in Liver Fibrosis: A Narrative Review

**DOI:** 10.3390/diagnostics16172694

**Published:** 2026-08-24

**Authors:** Arjuna Priyadarsin De Silva, Shashini Madushika Hathurusinghe, Madunil Anuk Niriella, Hithunadura Janaka De Silva

**Affiliations:** Department of Medicine, Faculty of Medicine, University of Kelaniya, Ragama 11010, Sri Lanka; shashini.m.hathurusinghe@gmail.com (S.M.H.); madunil.niriella@kln.ac.lk (M.A.N.); hjanakadesilva@gmail.com (H.J.D.S.)

**Keywords:** vibration-controlled transient elastography, point shear wave elastography, two-dimensional shear wave elastography, liver stiffness measurement, non-invasive assessment, chronic liver disease

## Abstract

Liver fibrosis staging plays a key role in the prognosis and management of chronic liver disease. Liver biopsy remains the reference standard for fibrosis assessment but is limited by its invasiveness and risk of complications, while magnetic resonance elastography, though also considered a gold standard, is constrained by cost and limited availability. Ultrasound-based elastography techniques—vibration-controlled transient elastography (VCTE), point shear wave elastography (pSWE), and two-dimensional shear wave elastography (2D SWE)—have emerged as accessible non-invasive alternatives. This narrative review aimed to summarize the principles, diagnostic performance, advantages, and limitations of these three techniques across a range of etiologies. A comprehensive literature search was conducted across Scopus, PubMed, Embase, CINAHL, the Cochrane Library, Informit, and the JBI Database of Systematic Reviews and Implementation Reports, covering January 2015 to February 2026. Search terms included “vibration-controlled transient elastography”, “point shear wave elastography”, “two-dimensional shear wave elastography”, “liver stiffness measurement”, “non-invasive assessment” and “chronic liver disease”. International guidelines, systematic reviews and meta-analyses, large observational cohort studies, and narrative reviews were included in the synthesis. VCTE, pSWE, and 2D SWE all demonstrated good diagnostic performance for detecting advanced fibrosis and cirrhosis across multiple etiologies. VCTE was the most extensively validated technique, with standardized cut-offs endorsed by major international guidelines. pSWE and 2D SWE showed comparable diagnostic accuracy and can be integrated into conventional ultrasound systems, enabling simultaneous structural and stiffness assessment, although validated cut-off values for these two techniques are not yet established in current guidelines, and absolute stiffness values are not directly interchangeable across techniques, manufacturers, or software versions. Obesity, inflammation, steatosis and recent food intake were identified as factors affecting measurement accuracy across all three techniques. Ultrasound-based elastography techniques offer effective, non-invasive alternatives to liver biopsy for fibrosis assessment. VCTE remains the most validated option despite cost and availability constraints, while pSWE and 2D SWE offer comparable accuracy with practical integration advantages. Future research should focus on standardizing cut-off values, addressing confounding factors, and combining elastography with biomarkers and AI-driven models.

## 1. Introduction

Chronic liver injury from a range of etiologies—including alcohol-related liver disease, metabolic dysfunction-associated steatotic liver disease (MASLD), autoimmune liver disease, and chronic hepatitis B and C—can lead to progressive fibrosis and cirrhosis, which carry substantial morbidity and mortality [1,2,3] and account for approximately 1.5 million deaths each year [1].

Assessing the severity of liver fibrosis and portal hypertension is important for the management and prognostication of chronic liver disease [4,5]. Fibrosis staging also informs treatment decisions, and appropriate treatment may halt or even reverse fibrosis progression [2]. Liver biopsy is the gold-standard investigation for fibrosis assessment [1]; however, its use has declined because it is invasive and painful and carries risks such as bleeding and infection [1,5,6,7]. Moreover, a biopsy samples only a tiny fraction of the liver (about 1/50,000), which can lead to diagnostic inaccuracy, particularly in early-stage fibrosis [7].

Non-invasive methods—serum biomarkers and elastography-based measures of liver stiffness—have emerged as alternatives that reduce the need for biopsy [3,5]. Serum biomarkers are particularly useful for identifying patients with advanced fibrosis, and their accuracy is limited in mild-to-moderate disease [7]. Elastography measures liver elasticity, which reflects the amount of fibrotic tissue present [2,4]. Ultrasound-based elastography techniques fall into two categories: strain elastography-based methods and shear wave elastography-based methods. Strain elastography-based methods include strain elastography and acoustic radiation force impulse (ARFI) strain elastography, whereas shear wave elastography-based methods include VCTE, pSWE, and 2D SWE [1,8].

Transient elastography (TE) for liver stiffness measurement (LSM) was first introduced in 2003. Image-guided US elastography systems, using standard probes with multiple transducer elements, were subsequently developed [2]. In 2008, the integration of shear wave elastography (SWE) into ultrasound systems further improved feasibility. Since then, a range of elastography technologies has emerged, together with classification frameworks such as that proposed by the European Federation of Societies for Ultrasound in Medicine and Biology (EFSUMB) [4]. Some studies have also combined elastography with serum markers to improve accuracy, because serum markers alone have low specificity for fibrosis and imaging techniques show measurement variability [7].

A comprehensive literature search was conducted across Scopus, PubMed, Embase, CINAHL, the Cochrane Library, Informit, and the JBI Database of Systematic Reviews and Implementation Reports, covering January 2015 to February 2026. The search combined the following MeSH terms and keywords: “Vibration-controlled transient elastography”, “Point shear wave elastography”, “Two-dimensional shear wave elastography”, “Liver stiffness measurement”, “Non-invasive assessment”, and “Chronic liver disease”.

Studies were eligible for inclusion if they were English-language, peer-reviewed reports involving human participants, published between January 2015 and February 2026, and comprised international guidelines, systematic reviews or meta-analyses, large observational cohort studies, or narrative reviews addressing VCTE, pSWE, or 2D SWE for liver fibrosis assessment. Case reports or case series with fewer than 10 patients, conference abstracts without an accompanying full-text publication, and non-English-language publications were excluded. Title and abstract screening were performed by one author, with borderline inclusion decisions verified by a second author. This narrative review draws on 63 sources, comprising international clinical guidelines, systematic reviews and meta-analyses, and observational studies identified through the search strategy described above; a formal record of the number of items retrieved, screened, and excluded at each stage was not maintained, consistent with the narrative (rather than systematic) design of this review. Overlapping primary studies across the cited meta-analyses were noted qualitatively where relevant, particularly in the comparative analyses in Section 4. Rather than formally deduplicated, and no formal risk-of-bias or AMSTAR-2 quality appraisal was undertaken; we consider this the appropriate level of methodological rigor for a narrative review and identify it as a limitation of the present work.

This review focuses on the three-shear wave elastography techniques—pSWE, VCTE, and 2D SWE—for the assessment of liver fibrosis. We provide a comprehensive overview of their principles, diagnostic performance, and comparative utility in clinical practice, and highlight their evolving role in the evaluation and monitoring of chronic liver disease.

## 2. Principles of Elastography Techniques

Liver elasticity reflects the amount of fibrotic tissue within the liver and is what elastography techniques quantify. Both ultrasound-based and MR-based methods are available; MR-based elastography has the higher diagnostic accuracy but is not used routinely because of its high cost and limited availability [1].

**Figure 1:**
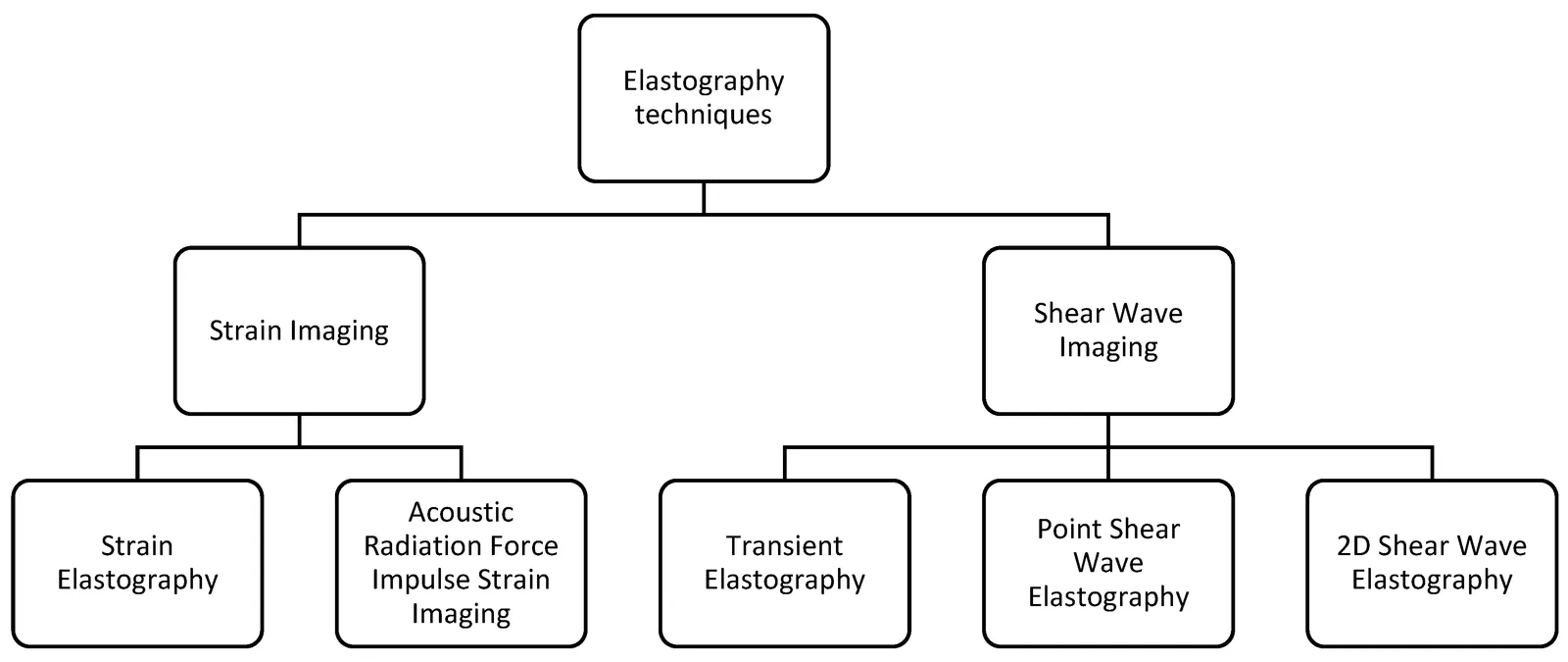
Types of ultrasound-based elastography techniques.

Ultrasound-based elastography techniques are of two broad types: strain-wave and shear-wave elastography. In strain elastography, the operator applies gentle manual compression with the probe and the system measures the resulting tissue strain or displacement on the principle that softer tissue deforms more than stiffer, fibrotic tissue; this yields a qualitative, colour-coded output. Shear-wave systems instead generate mechanical vibrations or ARFI excitations that produce shear waves within the liver (Figure 1). The speed of these shear waves is measured on the principle that they travel faster through stiffer tissue, so their velocity correlates with LSM [9].

### 2.1. Vibration Controlled Transient Elastography (VCTE)

TE is performed with a device that combines a low-frequency mechanical vibrator with an ultrasonic transducer [8]. Early TE systems had several limitations, including high measurement variability, owing to the use of a single transducer element and the absence of real-time image guidance, which made it difficult to target regions of homogeneous liver tissue accurately [2].

VCTE was developed as an advanced form of TE to overcome these limitations [2]. In VCTE, a mechanical vibrator generates low-frequency shear waves that propagate through the liver, and an ultrasound pulse-echo system measures their velocity; because shear wave velocity reflects tissue stiffness, VCTE provides a more accurate assessment of elasticity [8,9]. FibroScan^®^ is the most widely used VCTE device. VCTE can assess both liver and spleen stiffness and is regarded as a reliable method across various aetiologies [10,11]. It also derives the controlled attenuation parameter (CAP), which quantifies ultrasound attenuation attributable to hepatic fat content, whereas LSM quantifies tissue stiffness as a surrogate marker of fibrosis. CAP and LSM are complementary outputs of the same VCTE examination rather than interchangeable measures of the same underlying pathology, and joint interpretation of the two (see Section 9) is recommended for a complete assessment of steatosis and fibrosis.

VCTE is more accurate in advanced than in early fibrosis. Numerous studies and meta-analyses comparing VCTE with liver biopsy have found good accuracy for significant fibrosis and the best accuracy for the detection of cirrhosis [5].

Liver stiffness is reported in kilopascals (kPa), ranging from 1.5 to 75.0 kPa, with normal values around 5 kPa. Pediatric and adult probes are available while the adult probes operate at 50 Hz and come in different sizes (the M probe and the XL probe for a BMI greater than 30). Measurements are taken with the patient supine [9]. A dedicated splenic probe operating at 100 Hz is used to measure spleen stiffness (SSM).

### 2.2. 2D Shear Wave Elastography (2D SWE)

2D SWE uses ARFI excitations across multiple focal zones to generate shear waves and quantify their speed. The shear wave speed, measured in meters per second, is converted to kilopascals (kPa) using Young’s modulus [12]. It also produces a real-time, color-coded stiffness map over a relatively large area of the liver, typically about 2 cm × 2 cm, and stiffness can be sampled at multiple locations within the selected region of interest (ROI) [9]. Concurrent B-mode imaging helps identify the ROI and avoid artefacts [6,9].

### 2.3. Point Shear Wave Elastography (pSWE)

pSWE is likewise a validated method for detecting liver fibrosis of various aetiologies [11]. It uses ARFI excitation at a single focal location to generate shear waves and measure their speed in meters per second, which is then converted to kPa using Young’s modulus [13]. Measurements are taken over a smaller area (approximately 1.0 cm × 0.5 cm) than with VCTE or 2D SWE [8,9], with the ROI selected by the operator under B-mode guidance [2,8].

## 3. Stage-Wise Diagnostic Accuracy of Liver Elastography Techniques

Fibrosis staging is essential for the management of patients with chronic liver disease (CLD). In this review, fibrosis assessed by VCTE, pSWE, and 2D SWE is reported using the METAVIR system, with stages grouped as none/mild fibrosis (F0–F1), significant fibrosis (≥F2), advanced fibrosis (≥F3), and cirrhosis (F4).

### 3.1. VCTE

Multiple studies and meta-analyses have evaluated the diagnostic performance of VCTE across fibrosis stages. A 2024 meta-analysis of 19,199 patients with MASLD reported AUROCs of 0.83 for any fibrosis (≥F1), 0.83 for significant fibrosis, 0.87 for advanced fibrosis, and 0.94 for cirrhosis (Table 1), confirming that VCTE is highly reliable for detecting advanced fibrosis and cirrhosis [14]. In 230 patients with biopsy-proven MASLD, a systematic review and pooled analysis reported AUROCs of 0.82 for ≥F1, 0.87 for ≥F2, 0.84 for ≥F3, and 0.84 for F4 [15]. A meta-analysis of 6159 patients with MASLD found that, as a point-of-care test, VCTE had a pooled sensitivity of 76–89% and a pooled specificity of 67–73% across stages F1 to F4 [16].

VCTE has also been extensively evaluated in chronic hepatitis B. A meta-analysis of 4386 patients with chronic hepatitis B reported AUROCs of 0.91 and 0.93 for detecting advanced fibrosis and cirrhosis, respectively [20]. A meta-analysis of 8 studies and 2003 antiviral treatment-naïve patients with chronic hepatitis B found an AUROC of 0.81 for detecting significant fibrosis [21]. In that population of 2003 patients, VCTE had a pooled sensitivity of 0.78 and a summary specificity of 0.72 for significant fibrosis—consistent with earlier meta-analyses that reported sensitivities of 0.73–0.81 and specificities of 0.66–0.82 [20,21,22,23].

VCTE also performs well in autoimmune liver disease [24]. A meta-analysis of patients with autoimmune liver diseases (599 with primary biliary cholangitis [PBC], 388 with autoimmune hepatitis [AIH], and 151 with primary sclerosing cholangitis [PSC]) reported an AUROC greater than 0.85 across all fibrosis stages [24].

VCTE also compares favorably with serum biomarkers. In a meta-analysis of five non-invasive markers (LSM by VCTE, FIB-4, the MASLD Fibrosis Score, the AST-to-platelet ratio index, and the AST/ALT ratio), VCTE showed the highest diagnostic performance, with an AUROC of 0.85 [16].

Despite this good accuracy, the performance of VCTE depends on patient-related factors and operator experience, which may limit its reliability in some settings.

### 3.2. 2D SWE

2D SWE has also shown good accuracy for detecting liver fibrosis across multiple etiologies and stages [6,25]. In a large meta-analysis of 1134 patients, 2D SWE achieved AUROCs of 0.863, 0.906, and 0.855 for significant fibrosis and 0.929, 0.955, and 0.917 for cirrhosis in patients with hepatitis C, hepatitis B, and MASLD, respectively (Table 1) [26]. Uchikawa et al. reported AUROCs of 0.652, 0.732, and 0.761 for F1, F2, and F3, respectively, in 85 patients with fibrosis of mixed etiology [27].

More recent meta-analyses confirm the strong diagnostic performance of 2D SWE. A 2025 meta-analysis of 20 studies (2223 patients) reported pooled AUROCs of 0.82, 0.82, 0.86, and 0.89 for ≥F1, ≥F2, ≥F3, and F4, respectively [28]. Studies using the Canon ultrasound system reported an AUROC above 0.8 at every fibrosis stage in MASLD [29,30]. A meta-analysis of 5216 patients with chronic hepatitis B reported AUROCs of 0.93 for advanced fibrosis and 0.94 for cirrhosis [31], and a further meta-analysis of 11 studies (2623 patients with chronic hepatitis B) found good performance for significant fibrosis, with an AUROC of 0.92, a summary sensitivity of 88%, and a summary specificity of 83% [32].

Comparative studies show a strong correlation between 2D SWE liver stiffness and histological stage. In an evaluation of 13 non-invasive tools against liver biopsy in chronic hepatitis B, the strongest correlation was with 2D SWE (r = 0.779, *p* < 0.001), followed by VCTE (r = 0.719, *p* < 0.001) [7].

In patients with chronic hepatitis, LSM by 2D SWE correlated significantly with both fibrosis stage and FIB-4 score (*p* < 0.001). Diagnostic accuracy was high, with AUROCs of 0.956 for significant fibrosis (cut-off 8.2 kPa; sensitivity 92.6%, specificity 78.8%) and 0.978 for severe fibrosis (cut-off 10.1 kPa; sensitivity 92.9%, specificity 96.4%) (Table 2), underscoring its value for detecting fibrosis and monitoring treatment response [33].

Combining serum biomarkers with 2D SWE improves accuracy: one study reported a higher AUROC for the combination (0.889) than for 2D SWE alone (0.805) [34], and Zhang et al. similarly found that 2D SWE plus serum markers outperformed 2D SWE alone (AUROC 0.889 vs. 0.851) [7].

### 3.3. pSWE

Several meta-analyses have assessed the accuracy of pSWE for fibrosis staging. In a meta-analysis of 28 studies, Schambeck et al. reported a pooled sensitivity of 86% and specificity of 88% for significant fibrosis (Table 2), with an AUROC of 0.93, and concluded that pSWE is a good method for fibrosis assessment [35].

pSWE has also been compared directly with VCTE. A meta-analysis of nine pSWE studies (982 patients) and eleven VCTE studies (1753 patients) reported comparable performance in MASLD: pooled AUROCs for pSWE were 0.86, 0.94, and 0.95 for significant fibrosis, advanced fibrosis, and cirrhosis, versus 0.85, 0.92, and 0.94 for VCTE. Both techniques performed better for advanced fibrosis (≥F3) and cirrhosis (F4) than for significant fibrosis (≥F2), and the measurement failure rate was more than ten times higher with the VCTE M probe than with pSWE [8]. Losurdo et al. reported a pSWE AUROC of 0.98 for advanced fibrosis, falling to 0.88 for values below 6 kPa [36].

Further evidence supports the accuracy of pSWE in MASLD. A meta-analysis in MASLD reported an AUROC of 0.89 (sensitivity 85%, specificity 83%) for significant fibrosis (≥F2) and an AUROC of 0.94 (sensitivity 90%, specificity 95%) for cirrhosis [11].

In another study, pSWE showed significantly higher LSM in patients with MASLD (mean 10 ± 5.1 kPa) than in healthy individuals (mean 4.4 ± 0.7 kPa; *p* < 0.001), with stiffness rising across grades of fatty liver—supporting pSWE as a useful tool for fibrosis assessment, monitoring, and tracking disease progression [37].

Newer devices have also been evaluated. A study of a new pSWE device (X+ pSWE) reported AUROCs of 0.89 for significant fibrosis, 0.98 for severe fibrosis, and 0.90 for cirrhosis, using liver biopsy as the reference standard [38].

## 4. Comparative Diagnostic Accuracy of VCTE, pSWE, and 2D SWE

### 4.1. VCTE vs. pSWE vs. 2D SWE

The most recent meta-analysis (2026), which included 29 studies (4552 participants) and evaluated all US elastography techniques (VCTE, 2D SWE, and pSWE), reported a pooled AUROC of 0.932 (Table 3), a sensitivity of 88.8%, and a specificity of 89.2% (Table 4), confirming that US elastography is a highly effective non-invasive tool for fibrosis assessment [39].

Selvaraj et al., in a meta-analysis of 82 studies (14,609 patients with MASLD) comparing elastography techniques, found that pSWE had a superior summary AUROC to the other two techniques for significant fibrosis. AUROCs for significant fibrosis were 0.83, 0.75, and 0.86 for VCTE, 2D SWE, and pSWE, respectively; for advanced fibrosis, 0.85, 0.72, and 0.89; and for cirrhosis, 0.89, 0.88, and 0.90 [42].

A meta-analysis of 15 studies compared pSWE, 2D SWE, and VCTE. For significant fibrosis (≥F2), pooled sensitivity and specificity were 0.68 and 0.75 for pSWE, 0.85 and 0.72 for 2D SWE, and 0.79 and 0.73 for VCTE. On the basis of their 95% prediction intervals, VCTE and 2D SWE had similar overall accuracy for ≥F2 fibrosis, whereas pSWE had lower sensitivity but comparable specificity. For more advanced (≥F3) or early (≥F1) fibrosis, VCTE was more accurate than both SWE modalities, with pSWE performing least well. These findings suggest that 2D SWE and VCTE are preferable for non-invasive fibrosis assessment, while pSWE may be less sensitive at moderate stages [44].

Inconsistencies in the reported performance figures across studies likely reflect several sources of heterogeneity rather than true differences in technique performance alone. These include differences in the reference standard used (liver biopsy versus a composite or clinical diagnosis of fibrosis stage), fibrosis-stage prevalence within study cohorts (spectrum bias, whereby populations enriched for advanced disease can inflate apparent AUROC), the ultrasound device and manufacturer used (e.g., FibroScan for VCTE versus Aixplorer, Canon Aplio, or Esaote platforms for 2D SWE and pSWE), the probe selected (M versus XL probe, and differences in ARFI penetration depth), and study design (single- versus multicenter, and prospective versus retrospective). These factors should be borne in mind when comparing diagnostic performance figures across studies and are discussed further in Section 10.

### 4.2. 2D SWE vs. VCTE

A meta-analysis of 64 articles (13,046 patients with MASLD) found that SWE and MR elastography (MRE) outperformed other non-invasive tests, including VCTE. The summary sensitivity and specificity were 0.90 and 0.93 for SWE and 0.87 and 0.79 for VCTE, and the summary AUROCs were 0.88, 0.85, and 0.95 for the FibroScan M probe, XL probe, and SWE, respectively [43].

Herrmann et al., in a meta-analysis of 1134 patients with chronic hepatitis C (*n* = 379), hepatitis B (*n* = 400), and MASLD (*n* = 156), reported that 2D SWE had significantly higher diagnostic accuracy than VCTE, with AUROCs of 0.863, 0.906, and 0.855 for significant fibrosis (AUROC difference 0.022–0.084; *p* = 0.001) and 0.929, 0.955, and 0.917 for cirrhosis (AUROC difference 0.003–0.084; *p* = 0.022), respectively [26].

Another meta-analysis of 8 studies (1301 patients with chronic viral hepatitis) found comparable accuracy for advanced fibrosis and cirrhosis, with AUROCs of 0.93 (2D SWE) and 0.91 (VCTE) for advanced fibrosis and 0.97 and 0.95, respectively, for cirrhosis [3].

Karagiannakis et al. showed that LSM by 2D SWE correlated strongly with VCTE (*p* < 0.001) across etiologies, fibrosis stages, patient characteristics, and degrees of steatosis, supporting 2D SWE as a reliable alternative to VCTE, though absolute stiffness values and cut-offs are not directly interchangeable between the two techniques; the correlation held when distinguishing severe fibrosis (≥F3) and cirrhosis from lesser disease [5]. Zhang et al., comparing 2D SWE and VCTE in chronic hepatitis B and C, found similar diagnostic accuracy, although some studies report higher accuracy for 2D SWE [7]. Kobayashi et al. reported a strong positive correlation between 2D SWE and VCTE for LSM (r = 0.78, *p* < 0.001), suggesting that 2D SWE can substitute for VCTE, though absolute stiffness and cut-offs are not directly interchangeable across techniques, manufacturers, or software versions [6].

Zhang et al. also noted that, in chronic hepatitis B, VCTE and 2D SWE have similar accuracy, and that combining either technique with serum markers further improves performance, particularly for the advanced fibrosis stages that warrant clinical intervention [7].

In a study of 116 patients, 2D SWE showed excellent reliability (ICC 0.994) and correlated with histological fibrosis (r = 0.601, *p* < 0.001). AUROCs were 0.851 for ≥F2 fibrosis (cut-off 5.8 kPa) and 0.889 for cirrhosis (cut-off 9.6 kPa), comparable to VCTE, indicating that both are reliable non-invasive tools [45].

In another comparison of 2D SWE and VCTE in patients with liver disease, correlations varied by etiology: alcohol-related liver disease (ALD; r = 0.53), MASLD (r = 0.87), hepatitis B (r = 0.50), and hepatitis C (r = 0.76), all with *p* < 0.001 except ALD (*p* = 0.03) [46].

Del Valle et al., comparing VCTE with endoscopic ultrasound (EUS)-guided 2D SWE, found that EUS-2D SWE had higher diagnostic accuracy than VCTE, especially for assessing fibrosis in the left lobe [1].

The higher performance seen in some studies may reflect the ability of 2D SWE to provide real-time B-mode guidance and to sample a larger ROI than VCTE.

### 4.3. pSWE vs. 2D SWE

Mulazzani et al. found that the correlation between pSWE and 2D SWE was stronger at lower than at higher LSM values (≥15.2 kPa); this has limited clinical impact, however, because values above that threshold generally indicate cirrhosis, whereas the lower ranges are more relevant to treatment decisions. A comparable study of 26 patients using FibroScan reported good correlation with both pSWE and 2D SWE (*p* < 0.001) [4].

A meta-analysis of 13 studies (1527 patients with MASLD) compared pSWE and 2D SWE. For pSWE, summary AUROCs were 0.84, 0.91, and 0.94 for significant fibrosis (≥F2), advanced fibrosis (≥F3), and cirrhosis (F4), with sensitivities of 0.71, 0.81, and 0.81 and specificities of 0.83, 0.87, and 0.91. For 2D SWE, AUROCs were 0.83, 0.85, and 0.89, with sensitivities of 0.77, 0.80, and 0.92 and specificities of 0.76, 0.76, and 0.83. Overall, 2D SWE had slightly higher sensitivity and pSWE higher specificity, though the differences were not statistically significant [41].

A study assessing the correlation between a new pSWE device (X+ pSWE) and 2D SWE found good agreement between their LSM values (*p* < 0.001) [38]; however, 2D SWE had greater sensitivity and specificity than pSWE for ≥F2 and ≥F3 fibrosis when biopsy was the reference standard [38].

In a further comparison in MASLD, both techniques were accurate—particularly for significant fibrosis (AUROC 0.872 for pSWE vs. 0.965 for 2D SWE) and cirrhosis (0.886 vs. 0.994)—but 2D SWE showed higher accuracy, sensitivity (≥F2: 96.7% vs. 75.9%; F4: 100% vs. 76.5%), specificity (≥F2: 95.9% vs. 87.8%; F4: 95.1% vs. 91.8%), and reliability than pSWE [47].

### 4.4. VCTE vs. pSWE

In a comparison of elastography point quantification (EPQ) and VCTE in 353 patients with MASLD, EPQ was highly accurate, with AUROCs of 0.94 for significant fibrosis, 0.949 for advanced fibrosis, and 0.949 for cirrhosis and well-balanced sensitivity and specificity at the optimal cut-offs—comparable to VCTE, which also performed strongly for advanced fibrosis and cirrhosis. A strong positive correlation (Pearson’s r = 0.87) and high concordance (concordance correlation coefficient of 0.792) were observed between the two methods [11].

A meta-analysis of nine pSWE studies (982 patients) and eleven VCTE studies (1753 patients) reported similar performance in MASLD: AUROCs for pSWE were 0.86, 0.94, and 0.95 for significant fibrosis, advanced fibrosis, and cirrhosis, versus 0.85, 0.92, and 0.94 for VCTE. Measurement failure was more than ten times more frequent with the VCTE M probe. Overall, both provide accurate non-invasive staging, with pSWE showing lower failure rates [8].

In another comparison, pSWE correlated strongly with VCTE at higher fibrosis stages (r = 0.79) but weakly at lower stages (r = 0.19), and the correlation varied by etiology (r = 0.67 for HBV, 0.99 for ALD, 0.91 for HCV, 0.62 for MASLD, and 0.50 for AIH) [36].

Despite its favorable performance, pSWE samples only a small ROI, which may not adequately capture the heterogeneous distribution of liver fibrosis.

## 5. Elastography Techniques in Different Etiologies of Liver Fibrosis

Cut-off values reported in this review fall into three tiers of evidence with differing clinical applicability. Guideline-endorsed thresholds (e.g., EASL, AASLD, AGA, and Baveno VII) are validated across large, often multicenter cohorts and are appropriate for routine clinical decision-making. Meta-analysis-pooled “optimal” cut-offs, such as those summarized in Table 1, Table 2, Table 3 and Table 4, are derived statistically from pooled study data and are not yet formally endorsed by clinical guidelines. Single-study exploratory cut-off values are reported for completeness but derive from limited cohorts and should not be adopted directly in clinical practice without further validation. This hierarchy should be borne in mind when interpreting the cut-off values presented throughout this review.

### 5.1. Alcohol-Related Liver Disease

ALD is a major cause of liver-related morbidity and mortality, as affected patients often present with advanced disease and higher rates of complications. Non-invasive fibrosis testing is important, because early detection can prompt alcohol abstinence and halt progression. VCTE has the strongest evidence base in ALD, with excellent accuracy for advanced fibrosis (AUROC >0.90) and good accuracy for significant fibrosis (AUROC 0.85), although pSWE and 2D SWE are alternatives. According to European Association for the Study of the Liver (EASL) standards for ALD, VCTE values below 8–10 kPa rule out advanced fibrosis, whereas values ≥12–15 kPa rule it in [19].

The American Gastroenterological Association (AGA) 2017 guidelines recommend a cut-off of 12.5 kPa to diagnose cirrhosis in ALD [18].

### 5.2. MASLD

Early identification of patients at risk of progressive fibrosis in MASLD—particularly those at F2–F3 or with an LSM ≤8 kPa—allows timely initiation of therapy that can delay or prevent cirrhosis and reduce the global liver-related burden of MASLD [48,49]. Patients with MASLD and advanced fibrosis (F3–F4) are at higher risk of progression and adverse outcomes [26].

The American Association for the Study of Liver Diseases (AASLD) provides VCTE cut-offs of ≥12 kPa for advanced fibrosis and ≥20 kPa for cirrhosis and regards VCTE as a point-of-care test; for SWE, the advanced-fibrosis cut-offs are similar but less well validated [50].

The EASL 2021 guidelines note that non-invasive tests still have imperfect accuracy and that liver biopsy remains the gold standard in MASLD. pSWE and 2D SWE show similar accuracy but with less supporting data, whereas VCTE is the most extensively validated technique, with good performance for advanced fibrosis; MRE is the most accurate imaging method, though its availability and cost limit use. Per EASL, a VCTE value below 8 kPa rules out advanced fibrosis, while values above 12–15 kPa indicate advanced fibrosis [19]. In a meta-analysis of 63 studies (19,199 patients with MASLD), the optimal cut-off to rule out advanced fibrosis was 7.1–7.9 kPa (AUROC 0.90; sensitivity 89%, specificity 67%) [14].

The AGA 2017 guidelines made no recommendation on the use of VCTE in MASLD, reflecting an evidence gap at the time; this was addressed in subsequent guidelines [18].

Although guidelines recommend a modality-independent 8 kPa threshold as the low-risk cut-off for advanced fibrosis, a recent MASLD meta-analysis found that <8 kPa provided only 70–80% sensitivity for advanced fibrosis with 2D SWE and VCTE, and lower with pSWE. Higher sensitivity (~90%) was achievable with modality-specific cut-offs of 3 kPa (pSWE), 5 kPa (2D SWE), and 7 kPa (VCTE), while a 13 kPa threshold ruled in fibrosis (~90% specificity). These align with Society of Radiologists in Ultrasound (SRU) recommendations of ≤5 kPa to rule out and 5–13 kPa as indeterminate [28].

In a recent meta-analysis of 20 studies (2223 patients with MASLD), the optimal 2D SWE cut-offs were 6.43 kPa (≥F1), 8.17 kPa (≥F2), 9.42 kPa (≥F3), and 11.55 kPa (F4). Overall, 2D SWE showed excellent performance for fibrosis staging in MASLD, supporting the feasibility of standardized cut-offs [51].

### 5.3. Chronic Hepatitis B and C

Fibrosis staging is crucial in chronic viral hepatitis for guiding treatment and monitoring outcomes; a stage of ≥F2 is an indication to start antiviral therapy [7].

The AGA recommends VCTE cut-offs of 12.5 kPa for cirrhosis in chronic hepatitis C and 11.0 kPa for cirrhosis in chronic hepatitis B [18].

The EASL 2025 guidelines give VCTE cut-offs for significant fibrosis, advanced fibrosis, and cirrhosis in chronic hepatitis B of >7 kPa, >8 kPa, and >11 kPa, respectively [17]. The EASL 2020 guidelines set the hepatitis C cut-offs at 10 kPa for F3 and 13 kPa for F4, and the EASL 2021 guidelines strongly advise against applying untreated-HCV cut-offs to patients who have achieved a sustained virological response [19].

Some meta-analyses report that the optimal cut-off for significant fibrosis is lower in patients who have not received antiviral therapy (7.15 kPa) than in those who have (8.87 kPa) [33].

Kavak et al. reported that 2D SWE LSM values were highly accurate for assessing liver stiffness and could help avoid unnecessary biopsies; the technique may also aid follow-up and assessment of antiviral treatment response in chronic hepatitis B, with potential for routine monitoring [33].

pSWE performs better for assessing fibrosis in hepatitis C than in hepatitis B [19,36].

### 5.4. Autoimmune Disease

Fibrosis assessment is crucial in autoimmune liver disease for diagnosis, prognostication, and guiding and monitoring treatment; accurate tracking of fibrosis helps gauge treatment response and disease progression. Non-invasive methods such as VCTE are increasingly used because they are safe and suitable for repeated monitoring [24].

However, VCTE has recognized limitations in autoimmune-related cirrhosis, and different studies have proposed varying cut-offs derived from relatively small cohorts [24].

In PBC, advanced fibrosis carries a poor prognosis, so fibrosis assessment is essential for risk stratification. VCTE has high accuracy here (AUROC >0.85), and a cut-off of approximately 9.9–10.7 kPa is considered appropriate for detecting advanced fibrosis, consistent with European guidelines [24].

Although hepatic inflammation may overestimate LSM, particularly early after starting therapy, VCTE corresponds with the histological fibrosis stage and can be used to monitor disease progression [47].

The EASL 2021 guidelines note that 2D SWE shows promising results but limited disease-specific evidence, whereas pSWE has moderate accuracy [19]. The latest EASL 2025 guideline recommends VCTE for serial fibrosis assessment in AIH, with cut-offs of 9 kPa for severe fibrosis and 12.5–16 kPa for cirrhosis [52].

These inconsistent cut-offs highlight the need for etiology-specific thresholds, because LSM can be influenced by steatosis, inflammation, and cholestasis.

### 5.5. Pediatric and Obese Populations

Pediatric and obese populations remain underrepresented in the elastography literature. Validated liver-stiffness cut-offs for children are lacking, and normal stiffness values vary with growth and anthropometric change, complicating interpretation of pediatric LSM. A higher prevalence of increased subcutaneous fat in obese children further affects measurement reliability across all three techniques. Emerging evidence supports combining SWE with systemic inflammatory and metabolic markers to improve non-invasive characterization of hepatic steatosis and fibrosis risk in this population; for example, a study of 76 obese children and adolescents (compared with 44 healthy controls) found that SWE-measured LSM increased with hepatic steatosis grade and correlated significantly with systemic immune-inflammation indices [53].

## 6. Factors Affecting Diagnostic Performance

### 6.1. Patient-Related Factors

The diagnostic accuracy of non-invasive measures of liver fibrosis can be affected by several patient-related factors, including the location of the ROI, the skin-to-liver-capsule distance, and the body mass index (BMI), liver fat content, and the type of probe used [10]. The XL probe with lower-frequency waves was introduced to allow deeper tissue assessment [8,54]. The impact of obesity on pSWE measurements appears to be less pronounced [8].

Hepatic steatosis can also raise LSM independently of true fibrosis. This effect reflects a combination of fat infiltration itself, associated lobular inflammation and hepatocyte ballooning, and attenuation of the shear wave as it passes through fatty tissue, so an elevated stiffness reading in a steatotic liver does not necessarily indicate a correspondingly advanced fibrosis stage. This confound is only partly addressed for VCTE, which pairs the CAP with LSM in the same examination; pSWE and 2D SWE, outside of attenuation imaging-equipped platforms, generally lack an integrated fat-quantification counterpart, making steatosis-related overestimation of stiffness harder to identify with these two techniques [54].

This is particularly relevant in MASLD, where obesity and increased subcutaneous fat thickness are common and may interfere with reliable measurement [55]. Subcutaneous fat thickness disproportionately affects 2D SWE accuracy because of shear-wave attenuation with depth, in addition to its general effect on measurement reliability across techniques noted above.

### 6.2. Technique-Related Factors

The performance of VCTE can also be influenced by technique-related factors, such as the choice of probe (M vs. XL), the number and depth of valid measurements, the interquartile-range-to-median ratio of the readings, operator experience, and the interval since device calibration. Operator-applied transducer pressure can also pre-compress the underlying liver tissue and artificially elevate stiffness readings; a minimal-contact scanning technique, using adequate coupling gel and with the patient at rest and breathing freely, is recommended to avoid this artefact.

Diagnostic reliability is strongly operator-dependent; structured training, informed by EFSUMB guidance, of approximately 50 supervised examinations before independent unsupervised scanning is recommended and likely contributes to the inter-study heterogeneity in performance discussed elsewhere in this review.

Such factors can lead to over- or under-estimation of LSM and thereby affect clinical judgement when elastography is used diagnostically.

## 7. Reproducibility, Reliability and Feasibility

### 7.1. VCTE

VCTE shows excellent intra- and inter-observer reproducibility, with an intraclass correlation coefficient (ICC) of 0.98 in patients with liver disease of various etiologies [9].

In a large study of 7261 patients and more than 13,000 examinations, VCTE failed to obtain a measurement in 3% of cases and produced unreliable results in a further 15.8%—mainly because of obesity and operator inexperience—giving an overall applicability of 81%. Introduction of the XL probe improved applicability to over 95% in patients with MASLD. Reported unreliable-result rates of around 15–20% nonetheless point to a limitation in feasibility [9].

### 7.2. pSWE

pSWE has high repeatability and reproducibility, with consistent results on same-day measurement [13] and, in patients with liver disease of mixed etiology, high inter-observer (ICC 0.81–0.85) and intra-observer (ICC 0.89–0.90) reproducibility [55,56].

In a study comparing the reliability of pSWE and 2D SWE, failure rates were low for both (1% for pSWE and 5% for 2D SWE), and inter-observer agreement was significantly better for pSWE than for 2D SWE (ICC 0.915 vs. 0.829) [9].

### 7.3. 2D SWE

2D SWE also shows high intra-observer (ICC 0.93–0.95) and inter-observer (ICC 0.88) reproducibility, indicating good reliability for fibrosis assessment [13].

In a prospective cohort study of 2D SWE, 38 patients were examined separately to assess inter-observer reproducibility, yielding an ICC of 0.994 [45].

Lee et al. found that 2D SWE gave lower, less reliable results than pSWE in their study, possibly because the 2D SWE color map helped the operator identify a clean ROI; they also noted that fewer acquisitions were needed with 2D SWE than with pSWE to obtain reliable results [47].

## 8. Advantages and Limitations

Each technique has particular advantages and limitations that shape its clinical applicability.

### 8.1. VCTE

#### 8.1.1. Advantages

VCTE is well validated for fibrosis staging, with cut-offs established across many etiologies, particularly in compensated cirrhosis [11,47].

It is non-invasive and simple and provides rapid results, making it a useful point-of-care test for routine management and for assessing treatment response [9]. The EASL guidelines note its good reproducibility and high accuracy in cirrhosis, with an AUROC exceeding 0.9 [19].

VCTE is the most commonly used technique because it is a quick, simple bedside test requiring minimal training [9], and patient compliance is higher than with other methods; one large prospective multicenter study found that about 99% of patients returned for repeat VCTE, reflecting good acceptability [16]. That said, some studies report a progressive decline in attendance for follow-up assessment—Tapper et al. reported 91.4% at 3 months but 53% at 6 months [57].

#### 8.1.2. Disadvantages

VCTE has several disadvantages, chief among them inaccurate results in patients with a high BMI: a large skin-to-liver-capsule distance and obesity lead to overestimation [11], although the XL probe reduces this to some extent [2,8,22]. Perihepatic ascites, narrow intercostal spaces, and hepatic inflammation can also produce inaccurate results [9,11,57].

In one study monitoring disease progression, an apparent improvement from stage F4 to stage F2 fibrosis within a year—regarded by the investigators as biologically implausible—was attributed to overestimation of the initial stage by VCTE in a patient with class 3 obesity [58]. The device also requires recalibration every 6–12 months to maintain technical integrity [13].

EASL notes that inaccurate results may arise with food intake, high alcohol intake, hepatic inflammation, hepatic congestion, and extrahepatic cholestasis, and that the applicability of VCTE is even lower than that of serum biomarkers [19]. Absolute stiffness values are not directly comparable across manufacturers or software versions; serial or longitudinal monitoring of an individual patient should therefore use the same device, probe, and software version throughout follow-up, to avoid misinterpreting an artefactual change in stiffness as true disease progression or regression.

VCTE requires a dedicated transient-elastography device and cannot be performed on a conventional ultrasound system. Older devices provide no B-mode imaging and therefore cannot target the ROI, although newer machines now offer B-mode probes. Its use is largely confined to referral centers because of the high cost and the inaccurate results in obese patients [10].

Staufer et al. reported that VCTE gave inaccurate results in 20% of patients even with the XL probe and that the technique was not consistently feasible [10]; other studies likewise found that the XL probe does not always provide accurate results at higher BMI [5,57]. To address this, Echosens has introduced the “SmartExam” feature, which improves VCTE accuracy in obese patients [57].

### 8.2. pSWE

#### 8.2.1. Advantages

pSWE is validated for assessing liver fibrosis across multiple etiologies [11,58,59], and, unlike VCTE, both pSWE and 2D SWE can be integrated into a conventional abdominal ultrasound system [9].

pSWE also allows the operator to select the ROI and measurement depth directly on B-mode imaging, enabling precise positioning and avoidance of large vessels and other structures [9]. Its values are less affected by obesity and ascites, giving it higher applicability than VCTE [33].

According to the EASL guidelines, the diagnostic performance of pSWE is similar to that of VCTE in advanced fibrosis and cirrhosis [19].

#### 8.2.2. Disadvantages

As with VCTE, pSWE LSM is affected by food intake, high alcohol intake, hepatic congestion, higher degrees of steatosis, and hepatic inflammation [33,40].

pSWE cut-offs for significant and advanced fibrosis in MASLD have not yet been validated [11], nor have its cut-offs been validated across other etiologies in large-scale studies.

pSWE samples a small ROI, which may not represent the whole liver, and, unlike 2D SWE, it does not provide a color-coded elasticity map, limiting the visualization of tissue heterogeneity [9].

### 8.3. 2D SWE

#### 8.3.1. Advantages

2D SWE can be integrated into a conventional ultrasound system without specialized probes or machines, making it more accessible [5,6], and its B-mode capability allows selection of the ROI [6,7].

Unlike VCTE and pSWE, 2D SWE can interrogate a large area of the liver without additional probes, even in patients with obesity or ascites [5]. Kavak et al. note that, in contrast to liver biopsy, which samples a single point, 2D SWE captures a larger area and provides measurements at multiple points within the ROI [33].

The EASL 2021 guidelines note that 2D SWE also has good applicability, with higher performance for significant fibrosis and cirrhosis [19].

#### 8.3.2. Disadvantage

Ultrasound systems equipped with 2D SWE are expensive, limiting availability in many hospitals [7], and the technique requires specific operator training [5]. Guidelines have not yet introduced 2D SWE-specific criteria, and, compared with VCTE, 2D SWE has fewer supporting studies and less well-validated cut-offs [33]. EASL notes that, as with pSWE and VCTE, 2D SWE LSM can be affected by hepatic inflammation, food intake, high alcohol consumption, hepatic congestion, and extrahepatic cholestasis [19].

Absolute stiffness values are not directly comparable across manufacturers or software versions; serial or longitudinal monitoring of an individual patient should therefore use the same device, probe, and software version throughout follow-up to avoid misinterpreting an artefactual change in stiffness as true disease progression or regression.

## 9. Current Guidelines and Recommendations

International guidelines recommend VCTE for the non-invasive assessment of liver fibrosis in MASLD [11,44]. EFSUMB recommends VCTE, pSWE, and 2D SWE as first-line techniques for grading fibrosis and ruling out cirrhosis in MASLD, ALD, and chronic hepatitis B [36].

The EASL 2021 guidelines strongly recommend non-invasive tests—serum markers, fibrosis scores, elastography, and imaging—for risk-stratifying people with metabolic risk factors or harmful alcohol use to identify advanced fibrosis, as these perform better than clinical assessment alone [19].

EASL also identifies VCTE as useful for detecting patients at low risk of advanced fibrosis or cirrhosis [13]; its etiology-specific recommendations are summarized above.

The AASLD highlights the role of elastography in identifying patients with suspected MASLD referred from primary care who are at risk of steatohepatitis (MASH); it regards MRE and VCTE as effective for detecting advanced fibrosis in MASLD [44]. Recent evidence also suggests interpreting LSM together with the CAP value from the same examination to account for the effect of steatosis on stiffness and improve accuracy [10].

Compensated advanced chronic liver disease (cACLD), a term introduced by the Baveno VI consensus, spans the spectrum from advanced fibrosis to cirrhosis. The Baveno VII consensus introduced VCTE LSM cut-offs for cACLD: <10 kPa rules it out, 10–15 kPa suggests it, and >15 kPa makes it highly likely [60].

Baveno VII also notes that LSM is a practical, non-invasive tool for identifying patients with cACLD at high risk of clinically significant portal hypertension and decompensation, underscoring its prognostic value. It introduced a “rule of 5” for VCTE LSM (10–15–20–25 kPa), denoting progressively higher risks of decompensation and liver-related death, irrespective of etiology [60].

Although 2D SWE is widely used and highly accurate, most guidelines still regard VCTE as the reference method for LSM [5]. EASL reports that pSWE has accuracy comparable to VCTE, particularly in advanced fibrosis, but is less accurate in early fibrosis [19]. As noted in Section 5, these recommendations should be read against a hierarchy that distinguishes guideline-endorsed thresholds from study-derived pooled cut-offs and single-study exploratory values. International guidelines have not yet provided specific cut-offs for 2D SWE or pSWE. If shear wave elastography were validated as an alternative to VCTE, it could improve access to fibrosis evaluation [44].

## 10. Discussion

The introduction of elastography—VCTE, 2D SWE, and pSWE—has transformed the assessment of liver fibrosis, substantially reducing reliance on liver biopsy and enabling earlier recognition, risk stratification, and monitoring of treatment response.

VCTE remains the most validated technique, with guideline-endorsed cut-offs across etiologies, whereas 2D SWE and pSWE—despite comparable or superior accuracy—still lack standardized, guideline-endorsed thresholds. Real-world implementation is further limited by heterogeneous cut-offs across manufacturers and studies, high cost, restricted accessibility in resource-limited settings, and operator dependency, while confounders such as obesity, inflammation, and hepatic venous congestion continue to affect LSM values and hinder direct comparison across modalities. As a general principle governing interpretation throughout this review, absolute liver-stiffness values and their associated cut-offs are not necessarily interchangeable across elastography techniques, device manufacturers, or software versions, and numerical results should not be compared directly across platforms. For this reason, serial monitoring of an individual patient should use the same device and software version throughout.

## 11. Future Directions

Future research should evaluate how confounding factors affect LSM and how their impact can be minimized. Technological refinements—including improved probe sensitivity and advanced imaging—may overcome current technical barriers, especially in obese patients or those with difficult anatomy. Standardized protocols and operator training may reduce between-study inconsistency. Diagnostic models combining elastography with serum biomarkers and emerging AI-based tools should also be explored, as these may improve accuracy and longitudinal monitoring. In time, elastography is likely to become more accessible, automated, and standardized, and greater affordability and portability may extend its use into primary care and low-resource settings.

## 12. Conclusions

Non-invasive techniques such as VCTE, 2D SWE, and pSWE are important tools for assessing liver fibrosis across etiologies and have substantially reduced the need for liver biopsy, even though biopsy remains the gold standard. VCTE is the most extensively validated technique, with standard cut-offs provided by all major guidelines, though its high cost and limited availability remain constraints. 2D SWE and pSWE have shown accuracy comparable to VCTE, but validated stage-specific cut-offs are not yet included in current guidelines. Because both can be integrated into conventional ultrasound systems, the development of standardized cut-offs would enable simultaneous structural and stiffness assessment at reduced cost.

## Figures and Tables

**Table 1:** Pooled/representative AUROC values by technique, etiology, and fibrosis stage, with guideline-endorsed cut-offs where established.

Technique	Aetiology	Fibrosis Stage	AUROC	Guideline-Endorsed Cut-Off
VCTE	MASLD	F1	0.83	N/A (F1 not a guideline decision point)
VCTE	MASLD	≥F2	0.83	N/A (F2 not a guideline decision point)
VCTE	MASLD	≥F3	0.87	<8 kPa rules out; >12–15 kPa indicates advanced fibrosis [17]
VCTE	MASLD	F4	0.94	<8 kPa rules out; >12–15 kPa indicates advanced fibrosis [17]
VCTE	Chronic hepatitis B	≥F2 (significant)	—	>7 kPa [17]
VCTE	Chronic hepatitis B	≥F3 (advanced)	—	>8 kPa [17]
VCTE	Chronic hepatitis B	F4 (cirrhosis)	—	>11 kPa [17] ; 11.0 kPa [18]
VCTE	Chronic hepatitis C	≥F3 (advanced)	—	10 kPa [19]
VCTE	Chronic hepatitis C	F4 (cirrhosis)	—	13 kPa [19]; 12.5 kPa [18]
2D SWE	MASLD	≥F2	0.855	Not yet guideline-endorsed
2D SWE	MASLD	F4	0.917	Not yet guideline-endorsed
2D SWE	Chronic hepatitis B	≥F2	0.906	Not yet guideline-endorsed
2D SWE	Chronic hepatitis B	F4	0.955	Not yet guideline-endorsed
2D SWE	Chronic hepatitis C	≥F2	0.863	Not yet guideline-endorsed
2D SWE	Chronic hepatitis C	F4	0.929	Not yet guideline-endorsed
pSWE	Mixed etiology	≥F2	0.93	Not yet guideline-endorsed

AUROC, area under the receiver operating characteristic curve; MASLD, metabolic dysfunction-associated steatotic liver disease; VCTE, vibration-controlled transient elastography; 2D SWE, two-dimensional shear wave elastography; pSWE, point shear wave elastography. Fibrosis stages: F1 = mild fibrosis; F2 = moderate fibrosis; F3 = severe fibrosis; F4 = cirrhosis. ≥ denotes ‘at least’ the stated stage. Detailed numerical data from the individual meta-analyses underlying this table are provided in Supplementary Table S1.

**Table 2:** Representative sensitivity and specificity by technique, etiology, and fibrosis stage.

Technique	Etiology	Fibrosis Stage	Cut-Off (kPa)	Sensitivity	Specificity
VCTE	MASLD	F1	5–9.6	78%	75%
VCTE	MASLD	≥F2	4.8–16.4	79%	74%
VCTE	MASLD	≥F3	7.1–7.9	81%	79%
VCTE	MASLD	F4	6.9–20.1	91%	87%
2D SWE	MASLD	F1	6.4	76%	76%
2D SWE	MASLD	≥F2	8.2	76%	76%
2D SWE	MASLD	≥F3	9.4	79%	79%
2D SWE	MASLD	F4	11.6	82%	82%
2D SWE	Chronic hepatitis B	≥F2	7.91	88%	83%
pSWE	Mixed aetiology	≥F2	—	85.3%	89.5%

MASLD, metabolic dysfunction-associated steatotic liver disease; VCTE, vibration-controlled transient elastography; 2D SWE, two-dimensional shear wave elastography; pSWE, point shear wave elastography. Fibrosis stages: F1 = mild fibrosis; F2 = moderate fibrosis; F3 = severe fibrosis; F4 = cirrhosis. ≥ denotes ‘at least’ the stated stage. Detailed numerical data from the individual meta-analyses underlying this table are provided in Supplementary Table S2.

**Table 3:** Head-to-head comparative meta-analyses: AUROC at the key clinical decision point (≥F3, advanced fibrosis).

Technique	Meta-Analysis	Aetiology	Fibrosis Stage	AUROC
2D SWE	Zhou et al.[40]	Mixed	≥F3	0.95
pSWE	Zhou et al. [40]	Mixed	≥F3	0.90
pSWE	Xu et al. [41]	MASLD	≥F3	0.91
2D SWE	Xu et al. [41]	MASLD	≥F3	0.85
VCTE	Selvaraj et al. [42]	MASLD	≥F3	0.85
pSWE	Selvaraj et al. [42]	MASLD	≥F3	0.89
2D SWE	Selvaraj et al. [42]	MASLD	≥F3	0.72
pSWE	Jiang et al. [8]	MASLD	≥F3	0.94
VCTE	Jiang et al. [8]	MASLD	≥F3	0.92
VCTE	Xiao et al. [43]	MASLD	≥F3	0.85
2D SWE	Xiao et al. [43]	MASLD	≥F3	0.95

AUROC, area under the receiver operating characteristic curve; MASLD, metabolic dysfunction-associated steatotic liver disease; VCTE, vibration-controlled transient elastography; 2D SWE, two-dimensional shear wave elastography; pSWE, point shear wave elastography. Fibrosis stages:F3 = severe fibrosis ≥ denotes ‘at least’ the stated stage. Detailed numerical data across all reported fibrosis stages for these meta-analyses are provided in Supplementary Table S3.

**Table 4:** Head-to-head comparative meta-analyses: sensitivity and specificity at the key clinical decision point (≥F3, advanced fibrosis).

Technique	Meta-Analysis	Aetiology	Fibrosis Stage	Sensitivity	Specificity
2D SWE	Zhou et al. [40]	Mixed	≥F3	90%	87%
pSWE	Zhou et al. [40]	Mixed	≥F3	83%	83%
VCTE	Wilson et al. [28]	MASLD	≥F3	75%	75%
2D SWE	Wilson et al. [28]	MASLD	≥F3	77%	77%
pSWE	Wilson et al. [28]	MASLD	≥F3	78%	78%
pSWE	Xu et al. [41]	MASLD	≥F3	81%	87%
2D SWE	Xu et al. [41]	MASLD	≥F3	80%	76%
VCTE	Xiao et al. [43]	MASLD	≥F3	87%	79%
2D SWE	Xiao et al. [43]	MASLD	≥F3	90%	93%

MASLD, metabolic dysfunction-associated steatotic liver disease; VCTE, vibration-controlled transient elastography; 2D SWE, two-dimensional shear wave elastography; pSWE, point shear wave elastography. Fibrosis stages: F3 = severe fibrosis. ≥ denotes ‘at least’ the stated stage. Detailed numerical data across all reported fibrosis stages for these meta-analyses are provided in Supplementary Table S4.

## Data Availability

No new data were created or analyzed in this study. Data sharing is not applicable to this article.

## Supplementary Materials

The following supporting information can be downloaded at: https://www.mdpi.com/article/10.3390/diagnostics16172694/s1, Table S1: Comparison of meta-analyses on elastography techniques: AUROC values for stages of liver fibrosis; Table S2: Comparison of meta-analyses on elastography techniques: Summary sensitivity and specificity for stages of liver fibrosis; Table S3: Comparison of meta-analyses that compared two or more elastography techniques: AUROC values for stages of liver fibrosis; Table S4: Comparison of meta-analyses that compared two or more elastography techniques: Summary sensitivity and specificity for stages of liver fibrosis.

## References

[1] Del Valle R., Cunto D., Puga-Tejada M., Egas-Izquierdo M., Arevalo-Mora M., Oleas R., Alcivar-Vasquez J., Dal Bello F., Pitanga-Lukashok H., Baquerizo-Burgos J. (2025). Comparative analysis of vibration-controlled transient elastography and EUS-shear wave elastography for liver stiffness measurement in cirrhosis. Endosc. Ultrasound.

[2] Bergsma S., van Gent M., Dam-Vervloet A.J., Lagerweij M.C.M., van der Wouden E.J., Nijholt I.M., Boomsma M.F., Poot L. (2024). Image-guided point-shear-wave elastography: A valid and reliable technique for liver fibrotic staging. J. Ultrasound.

[3] Luo Q.-T., Zhu Q., Zong X.-D., Li M.-K., Yu H.-S., Jiang C.-Y., Liao X. (2022). Diagnostic performance of transient elastography versus two-dimensional shear wave elastography for liver fibrosis in chronic viral hepatitis: Direct comparison and a meta-analysis. Biomed. Res. Int..

[4] Mulazzani L., Salvatore V., Ravaioli F., Allegretti G., Matassoni F., Granata R., Ferrarini A., Stefanescu H., Piscaglia F. (2017). Point shear wave ultrasound elastography with Esaote compared to real-time 2D shear wave elastography with supersonic imagine for the quantification of liver stiffness. J. Ultrasound.

[5] Karagiannakis D.S., Voulgaris T., Angelopoulos T., Ioannidou P., Cholongitas E., Vlachogiannakos J., Papatheodoridis G.V. (2021). Comparative utility of transient and 2D shear wave elastography for the assessment of liver fibrosis in clinical practice. J. Digit. Imaging.

[6] Kobayashi T., Nakatsuka T., Sato M., Soroida Y., Hikita H., Gotoh H., Iwai T., Tateishi R., Kurano M., Fujishiro M. (2025). Diagnostic performance of two-dimensional shear wave elastography and attenuation imaging for fibrosis and steatosis assessment in chronic liver disease. J. Med. Ultrasound.

[7] Zhang Y., Yang J., Wan Q., Gan L., Liu J. (2026). Non-invasive assessment of liver fibrosis staging in chronic hepatitis B patients: Combining two-dimensional shear wave elastography with serum indicators. Front. Med..

[8] Jiang W., Huang S., Teng H., Wang P., Wu M., Zhou X., Ran H. (2018). Diagnostic accuracy of point shear wave elastography and transient elastography for staging hepatic fibrosis in patients with non-alcoholic fatty liver disease: A meta-analysis. BMJ Open.

[9] Vranić L., Nadarević T., Štimac D., Fraquelli M., Manzotti C., Casazza G., Colli A. (2022). Liver and spleen stiffness as assessed by vibration-controlled transient elastography for diagnosing clinically significant portal hypertension in comparison with other elastography-based techniques in adults with chronic liver disease. Cochrane Database Syst. Rev..

[10] Staufer K., Halilbasic E., Spindelboeck W., Eilenberg M., Prager G., Stadlbauer V., Posch A., Munda P., Marculescu R., Obermayer-Pietsch B. (2019). Evaluation and comparison of six noninvasive tests for prediction of significant or advanced fibrosis in nonalcoholic fatty liver disease. United Eur. Gastroenterol. J..

[11] Bauer D.J.M., Matic V., Mare R., Maiocchi L., Chromy D., Müllner-Bucsics T., Mandorfer M., Mustapic S., Sporea I., Ferraioli G. (2023). Point shear wave elastography by ElastPQ for fibrosis screening in patients with MASLD: A prospective, multicenter comparison to vibration-controlled elastography. Ultraschall. Med..

[12] Lee S., Choi Y.H., Cho Y.J., Lee S.B., Cheon J.-E., Kim W.S., Ko J.S., Koh J., Kang G.H. (2021). The usefulness of noninvasive liver stiffness assessment using shear-wave elastography for predicting liver fibrosis in children. BMC Med. Imaging.

[13] Honda Y., Yoneda M., Imajo K., Nakajima A. (2020). Elastography techniques for the assessment of liver fibrosis in non-alcoholic fatty liver disease. Int. J. Mol. Sci..

[14] Chon Y.E., Jin Y.-J., An J., Kim H.Y., Choi M., Jun D.W., Kim M.N., Han J.W., Lee H.A., Yu J.H. (2024). Optimal cut-offs of vibration-controlled transient elastography and magnetic resonance elastography in diagnosing advanced liver fibrosis in patients with nonalcoholic fatty liver disease: A systematic review and meta-analysis. Clin. Mol. Hepatol..

[15] Hsu C., Caussy C., Imajo K., Chen J., Singh S., Kaulback K., Le M.D., Hooker J., Tu X., Bettencourt R. (2019). Magnetic resonance vs transient elastography analysis of patients with nonalcoholic fatty liver disease: A systematic review and pooled analysis of individual participants. Clin. Gastroenterol. Hepatol..

[16] Sarkar Das T., Meng X., Abdallah M., Bilal M., Sarwar R., Shaukat A. (2024). An assessment of the feasibility, patient acceptance, and performance of point-of-care transient elastography for metabolic-dysfunction-associated steatotic liver disease (MASLD): A systematic review and meta-analysis. Diagnostics.

[17] Cornberg M., Sandmann L., Jaroszewicz J. (2025). EASL Clinical Practice Guidelines on the management of hepatitis B virus infection. J. Hepatol..

[18] Lim J.K., Flamm S.L., Singh S., Falck-Ytter Y.T., Clinical Guidelines Committee of the American Gastroenterological Association (2017). American Gastroenterological Association Institute guideline on the role of elastography in the evaluation of liver fibrosis. Gastroenterology.

[19] European Association for the Study of the Liver (2021). EASL clinical practice guidelines on non-invasive tests for evaluation of liver disease severity and prognosis. J. Hepatol..

[20] Li Y., Huang Y.-S., Wang Z.-Z., Yang Z.-R., Sun F., Zhan S.-Y., Liu X.-E., Zhuang H. (2016). Systematic review with meta-analysis: The diagnostic accuracy of transient elastography for the staging of liver fibrosis in patients with chronic hepatitis B. Aliment Pharmacol. Ther..

[21] Kim M.N., An J., Kim E.H., Kim H.Y., Lee H.A., Yu J.H., Jin Y.-J., Chon Y.E., Kim S.U., Jun D.W. (2024). Vibration-controlled transient elastography for significant fibrosis in treatment-naïve chronic hepatitis B patients: A systematic review and meta-analysis. Clin. Mol. Hepatol..

[22] Li M., Wang S., Wang X., Li Y., Wu B. (2022). Diagnostic performance of elastography on liver fibrosis in antiviral treatment-naive chronic hepatitis B patients: A meta-analysis. Gastroenterol. Rep..

[23] Chon Y.E., Choi E.H., Song K.J., Park J.Y., Kim D.Y., Han K.-H., Chon C.Y., Ahn S.H., Kim S.U. (2012). Performance of transient elastography for the staging of liver fibrosis in patients with chronic hepatitis B: A meta-analysis. PLoS ONE.

[24] An J., Chon Y.E., Kim G., Kim M.N., Kim H.Y., Lee H.A., Yu J.H., Choi M., Jun D.W., Kim S.U. (2024). Diagnostic accuracy of vibration-controlled transient elastography for staging liver fibrosis in autoimmune liver diseases: A systematic review and meta-analysis. Clin. Mol. Hepatol..

[25] Zhang W., Zhu Y., Zhang C., Ran H. (2019). Diagnostic accuracy of 2-dimensional shear wave elastography for the staging of liver fibrosis: A meta-analysis. J. Ultrasound Med..

[26] Herrmann E., de Lédinghen V., Cassinotto C., Chu W.C.-W., Leung V.Y.-F., Ferraioli G., Filice C., Castera L., Vilgrain V., Ronot M. (2018). Assessment of biopsy-proven liver fibrosis by two-dimensional shear wave elastography: An individual patient data-based meta-analysis. Hepatology.

[27] Uchikawa S., Kawaoka T., Fujino H., Ono A., Nakahara T., Murakami E., Yamauchi M., Miki D., Imamura M., Aikata H. (2022). The effect of the skin-liver capsule distance on the accuracy of ultrasound diagnosis for liver steatosis and fibrosis. J. Med. Ultrason..

[28] Wilson M.P., Singh R., Mehta S., Murad M.H., Fung C., Low G. (2025). Comparing FIB-4, VCTE, pSWE, 2D-SWE, and MRE thresholds and diagnostic accuracies for detecting hepatic fibrosis in patients with MASLD: A systematic review and meta-analysis. Diagnostics.

[29] Sugimoto K., Moriyasu F., Oshiro H., Takeuchi H., Abe M., Yoshimasu Y., Kasai Y., Sakamaki K., Hara T., Itoi T. (2020). The role of multiparametric US of the liver for the evaluation of nonalcoholic steatohepatitis. Radiology.

[30] Lee D.H., Cho E.J., Bae J.S., Lee J.Y., Yu S.J., Kim H., Lee K.B., Han J.K., Choi B.I. (2021). Accuracy of two-dimensional shear wave elastography and attenuation imaging for evaluation of patients with nonalcoholic steatohepatitis. Clin. Gastroenterol. Hepatol..

[31] Dong B., Lyu G., Chen Y., Lin G., Wang H., Qin R., Gu J. (2021). Comparison of two-dimensional shear wave elastography, magnetic resonance elastography, and three serum markers for diagnosing fibrosis in patients with chronic hepatitis B: A meta-analysis. Expert. Rev. Gastroenterol. Hepatol..

[32] Wei H., Jiang H.Y., Li M., Zhang T., Song B. (2020). Two-dimensional shear wave elastography for significant liver fibrosis in patients with chronic hepatitis B: A systematic review and meta-analysis. Eur. J. Radiol..

[33] Kavak S., Kaya S., Senol A., Sogutcu N. (2022). Evaluation of liver fibrosis in chronic hepatitis B patients with 2D shear wave elastography with propagation map guidance: A single-centre study. BMC Med. Imaging.

[34] Boursier J., Hagström H., Traussnigg S., Semmler G., Pini P., Moussata D., Fouchard-Hubert I., Leroy V., Moessner B., Ekstedt M. (2022). Diagnostic accuracy of non-invasive tests for advanced fibrosis in patients with MASLD: An individual patient data meta-analysis. Gut.

[35] Schambeck J.P.L., Forte G.C., Gonçalves L.M., Stuker G., Kotlinski J.B.F., Tramontin G., Altmayer S., Watte G., Hochhegger B. (2023). Diagnostic accuracy of magnetic resonance elastography and point-shear wave elastography for significant hepatic fibrosis screening: Systematic review and meta-analysis. PLoS ONE.

[36] Losurdo G., Ditonno I., Novielli D., Celiberto F., Iannone A., Castellaneta A., Dell’Aquila P., Ranaldo N., Rendina M., Barone M. (2024). Comparison of transient elastography and point shear wave elastography for analysis of liver stiffness: A prospective study. Diagnostics.

[37] Patil P.V., Julakanti S., Dhadve R.U. (2024). Point shear wave elastography for assessment of liver stiffness in normal individuals and in patients with non-alcoholic fatty liver disease. Cureus.

[38] Garcovich M., Paratore M., Riccardi L., Zocco M.A., Ainora M.E., Mingrone G., Gasbarrini A., Pompili M. (2023). Correlation between a new point-shear wave elastography device (X+pSWE) with liver histology and 2D-SWE (SSI) for liver stiffness quantification in chronic liver disease. Diagnostics.

[39] Gilani S.A., Farooq S.M.Y., Moazzam M., Hanif A., Uzair M., Iyaz M., Niazi Y. (2026). Ultrasound elastography to assess liver fibrosis: A systematic review and meta-analysis. J. Diagn. Med. Sonogr..

[40] Zhou X., Rao J., Wu X., Deng R., Ma Y. (2021). Comparison of 2-D shear wave elastography and point shear wave elastography for assessing liver fibrosis. Ultrasound Med. Biol..

[41] Xu X., Zhang Y., Zhu Q., Xie Y., Zhou Y., Dong B., Zhang C. (2025). Diagnostic accuracy of two-dimensional shear wave elastography and point shear wave elastography in identifying different stages of liver fibrosis in patients with metabolic dysfunction-associated steatotic liver disease: A meta-analysis. Biomol. Biomed..

[42] Selvaraj E.A., Mózes F.E., Jayaswal A.N.A., Zafarmand M.H., Vali Y., Lee J.A., Levick C.K., Young L.A.J., Palaniyappan N., Liu C.-H. (2021). Diagnostic accuracy of elastography and magnetic resonance imaging in patients with MASLD: A systematic review and meta-analysis. J. Hepatol..

[43] Xiao G., Zhu S., Xiao X., Yan L., Yang J., Wu G. (2017). Comparison of laboratory tests, ultrasound, or magnetic resonance elastography to detect fibrosis in patients with nonalcoholic fatty liver disease: A meta-analysis. Hepatology.

[44] Yamaguchi R., Oda T., Nagashima K. (2025). Comparison of the diagnostic accuracy of shear wave elastography with transient elastography in adult nonalcoholic fatty liver disease: A systematic review and network meta-analysis of diagnostic test accuracy. Abdom. Radiol..

[45] Yoo H.W., Kim S.G., Jang J.Y., Yoo J.J., Jeong S.W., Kim Y.S., Kim B.S. (2022). Two-dimensional shear wave elastography for assessing liver fibrosis in patients with chronic liver disease: A prospective cohort study. Korean J. Intern. Med..

[46] Ayonrinde O.T., Zelesco M., Welman C.J., Abbott S., Adris N. (2022). Clinical relevance of shear wave elastography compared with transient elastography and other markers of liver fibrosis. Intern. Med. J..

[47] Lee S.M., Ha H.I., Lee I.J., Lee K., Lee J.W., Park J.W., Kim S.-E., Kwon M.J., Choe J.-Y., Yoon S.-Y. (2023). Comparison between two-dimensional and point shear wave elastography techniques in evaluating liver fibrosis using histological staging as the reference standard: A prospective pilot study. Diagnostics.

[48] Mettananda C., Egodage T., Dantanarayana C., Fernando R., Ranaweera L., Luke N., Ranawaka C., Kottahachchi D., Pathmeswaran A., de Silva H.J. (2023). Identification of patients with type 2 diabetes with non-alcoholic fatty liver disease who are at increased risk of progressing to advanced fibrosis: A cross-sectional study. BMJ Open.

[49] Hashemi S.A., Alavian S.M., Gholami-Fesharaki M. (2016). Assessment of transient elastography (FibroScan) for diagnosis of fibrosis in non-alcoholic fatty liver disease: A systematic review and meta-analysis. Casp. J. Intern. Med..

[50] Rinella M.E., Neuschwander-Tetri B.A., Siddiqui M.S., Abdelmalek M.F., Caldwell S., Barb D., Bamhaoud S., Terrault N.A., Anstee Q.M., Sanyal A.J. (2023). AASLD practice guidance on the clinical assessment and management of nonalcoholic fatty liver disease. Hepatology.

[51] Indre M.-G., Leucuta D.-C., Lupsor-Platon M., Turco L., Ferri S., Hashim A., Orasan O.H., Procopet B., Pasulo L., Ravaioli F. (2025). Diagnostic accuracy of 2D-SWE ultrasound for liver fibrosis assessment in MASLD: A multilevel random effects model meta-analysis. Hepatology.

[52] European Association for the Study of the Liver (2025). EASL Clinical Practice Guidelines on the management of autoimmune hepatitis. J. Hepatol..

[53] Akçiçek M., Dağ N. (2025). Evaluation of hepatic steatosis in obese children and adolescents using immune-inflammatory markers and shear wave elastography. J. Ultrasound.

[54] Bauer D.J., De Silvestri A., Maiocchi L., Raimondi A., Mare R., Mandorfer M., Reiberger T. (2025). Understanding confounding factors allows for accurate interpretation of liver stiffness measurements by ElastQ, a novel 2D shear wave elastography technique. Ultraschall. Med..

[55] Han A., Labyed Y., Sy E.Z., Boehringer A.S., Andre M.P., Erdman J.W., Loomba R., Sirlin C.B., O’Brien W.D. (2018). Inter-sonographer reproducibility of quantitative ultrasound outcomes and shear wave speed measured in the right lobe of the liver in adults with known or suspected non-alcoholic fatty liver disease. Eur. Radiol..

[56] Balakrishnan M., Souza F., Muñoz C., Augustin S., Loo N., Deng Y., Ciarleglio M., Garcia-Tsao G. (2016). Liver and spleen stiffness measurements by point shear wave elastography via acoustic radiation force impulse: Intraobserver and interobserver variability and predictors of variability in a US population. J. Ultrasound Med..

[57] Tapper E.B., Challies T., Nasser I., Afdhal N.H., Lai M. (2016). The performance of vibration controlled transient elastography in a US cohort of patients with nonalcoholic fatty liver disease. Am. J. Gastroenterol..

[58] Woodard J.S., Velji-Ibrahim J., Alden J., Abrams G.A. (2024). VCTE overestimates liver fibrosis due to abdominal-truncal adiposity and not hepatic steatosis: A case report. Case Rep. Gastrointest. Med..

[59] Bucsics T., Grasl B., Ferlitsch A., Schwabl P., Mandorfer M., Zinober K., Stern R., Chromy D., Scheiner B., Sieghart W. (2018). Point shear wave elastography for non-invasive assessment of liver fibrosis in patients with viral hepatitis. Ultrasound Med. Biol..

[60] de Franchis R., Bosch J., Garcia-Tsao G., Reiberger T., Ripoll C., Yoshiji H., Baveno VII Faculty (2022). Baveno VII—Renewing consensus in portal hypertension. J. Hepatol..

