# Supplementary material for: The Use of Ultrasound-Based Elastography Techniques in Liver Fibrosis: A Narrative Review
Source: Diagnostics (Basel). 2026 Aug 24;16(17):2694. doi: 10.3390/diagnostics16172694 (PMC13565457; doi:10.3390/diagnostics16172694)
Supplement: Supplementary file 1 [file diagnostics-16-02694-s001.zip › diagnostics-4490179-supplementary.pdf]

Table S1. Comparison of meta-analyses on elastography techniques: AUROC values for stages of liver fibrosis

Technique	Meta-analysis	Aetiology	Fibrosis Stage	AUROC
**VCTE**	Mózes et al.	MASLD	≥F3	0.85
	Chon et al.	MASLD	F1	0.83
			≥F2	0.83
			≥F3	0.87
			F4	0.94
	Hsu et al.	MASLD	F1	0.82
			≥F2	0.87
			≥F3	0.84
			F4	0.84
	Hashemi et al.	MASLD	F1	0.776–0.92
			≥F2	0.78–0.99
			≥F3	0.7–1
			F4	0.94–0.99
**2D SWE**	Indre et al.	MASLD	F1	0.82
			≥F2	0.82
			≥F3	0.86
			F4	0.89
	Zhang et al.	Mixed	≥F2	0.81
			≥F3	0.77
			F4	0.84
	Wei et al.	Chronic hepatitis B	≥F2	0.92
	Herrmann et al.	MASLD	≥F2	0.855
			F4	0.917

	Herrmann et al.	Chronic hepatitis B	≥F2	0.906
			F4	0.955
	Herrmann et al.	Chronic hepatitis C	≥F2	0.863
			F4	0.929
**pSWE**	Schambeck et al.	Mixed	≥F2	0.93

AUROC, area under the receiver operating characteristic curve; MASLD, metabolic dysfunction-associated steatotic liver disease; VCTE, vibration-controlled transient elastography.
Fibrosis stages: F1 = mild fibrosis; F2 = moderate fibrosis; F3 = severe fibrosis; F4 = cirrhosis. ≥ denotes 'at least' the stated stage.

Table S2. Comparison of meta-analyses on elastography techniques: Summary sensitivity and specificity for stages of liver fibrosis

Technique	Meta-analysis	Aetiology	Fibrosis Stage	Cut-off (kPa)	Sensitivity	Specificity
VCTE	Mózes et al.	MASLD	≥F3	9.1	77%	78%
	Chon et al.	MASLD	F1	5–9.6	78%	75%
			≥F2	4.8–16.4	79%	74%
			≥F3	7.1–7.9	81%	79%
			F4	6.9–20.1	91%	87%
	Hsu et al.	MASLD	F1	6.2	65.6%	67.1%
			≥F2	7.6	76.3%	79.6%
			≥F3	8.8	77.2%	78%
			F4	11.8	80%	81%
	Hashemi et al.	MASLD	F1	5.35–5.9	83.7%	78.2%
			≥F2	5.35–7.4	87.5%	78.4%
			≥F3	7.1–12.85	93.7%	91.1%
			F4	10.3–17.5	96.2%	92.2%
2D SWE	Indre et al.	MASLD	F1	6.4	76%	76%
			≥F2	8.2	76%	76%
			≥F3	9.4	79%	79%
			F4	11.6	82%	82%
	Zhang et al.	Mixed	≥F2	–	85%	79%
			≥F3	–	90%	85%
			F4	–	89%	92%

	Wei et al.	Chronic hepatitis B	≥F2	7.91	88%	83%
**pSWE**	Schambeck et al.	Mixed	≥F2	–	85.26%	89.46%

AUROC, area under the receiver operating characteristic curve; MASLD, metabolic dysfunction-associated steatotic liver disease; VCTE, vibration-controlled transient elastography.

Fibrosis stages: F1 = mild fibrosis; F2 = moderate fibrosis; F3 = severe fibrosis; F4 = cirrhosis. ≥ denotes 'at least' the stated stage.

Table S3. Comparison of meta-analyses that compared two or more elastography techniques: AUROC values for stages of liver fibrosis

Technique	Meta-analysis	Aetiology	Fibrosis Stage	AUROC
**2D SWE**	Zhou et al.	Mixed	≥F2	0.89
			≥F3	0.95
			F4	0.94
**pSWE**	Zhou et al.	Mixed	≥F2	0.85
			≥F3	0.90
			F4	0.91
**pSWE**	Xu et al.	MASLD	≥F2	0.84
			≥F3	0.91
			F4	0.94
**2D SWE**	Xu et al.	MASLD	≥F2	0.83
			≥F3	0.85
			F4	0.89
**VCTE**	Selvaraj et al.	MASLD	≥F2	0.83
			≥F3	0.85

			F4	0.89
**pSWE**	Selvaraj et al.	MASLD	≥F2	0.86
			≥F3	0.89
			F4	0.90
**2D SWE**	Selvaraj et al.	MASLD	≥F2	0.75
			≥F3	0.72
			F4	0.88
**pSWE**	Jiang et al.	MASLD	≥F2	0.86
			≥F3	0.94
			≥F4	0.95
**VCTE**	Jiang et al.	MASLD	≥F2	0.85
			≥F3	0.92
			F4	0.94
**VCTE**	Xiao et al.	MASLD	≥F3	0.85
**2D SWE**	Xiao et al.	MASLD	≥F3	0.95

AUROC, area under the receiver operating characteristic curve; MASLD, metabolic dysfunction-associated steatotic liver disease; VCTE, vibration-controlled transient elastography.
Fibrosis stages: F1 = mild fibrosis; F2 = moderate fibrosis; F3 = severe fibrosis; F4 = cirrhosis. ≥ denotes 'at least' the stated stage.

Table S4. Comparison of meta-analyses that compared two or more elastography techniques: Summary sensitivity and specificity for stages of liver fibrosis

Technique	Meta-analysis	Aetiology	Fibrosis Stage	Cut-off (kPa)	Sensitivity	Specificity
**2D SWE**	Zhou et al.	Mixed	≥F2	–	84%	81%
			≥F3	–	90%	87%
			F4	–	89%	87%
**pSWE**	Zhou et al.	Mixed	≥F2	–	76%	79%
			≥F3	–	83%	83%
			F4	–	85%	84%
**VCTE**	Wilson et al.	MASLD	≥F2	8.48	72%	72%
			≥F3	9.74	75%	75%
**2D SWE**	Wilson et al.	MASLD	≥F2	8.31	74%	74%
			≥F3	9.58	77%	77%
**pSWE**	Wilson et al.	MASLD	≥F2	5.37	74%	74%
			≥F3	6.43	78%	78%
**pSWE**	Xu et al.	MASLD	≥F2	–	71%	83%

			≥F3	–	81%	87%
			F4	–	81%	91%
**2D SWE**	Xu et al.	MASLD	≥F2	–	77%	76%
			≥F3	–	80%	76%
			F4	–	92%	83%
**VCTE**	Xiao et al.	MASLD	≥F3	–	87%	79%
**2D SWE**	Xiao et al.	MASLD	≥F3	–	90%	93%
**VCTE**	Jiang et al.	MASLD	≥F2	–	77%	80%
			≥F3	–	79%	89%
			F4	–	90%	91%
**pSWE**	Jiang et al.	MASLD	≥F2	–	70%	84%
			≥F3	–	89%	88%
			F4	–	89%	91%
**VCTE**	Bota et al.	Mixed	≥F2	–	78%	84%
			F4	–	89%	87%
**pSWE**	Bota et al.	Mixed	≥F2	–	74%	83%
			F4	–	87%	87%

AUROC, area under the receiver operating characteristic curve; MASLD, metabolic dysfunction-associated steatotic liver disease; VCTE, vibration-controlled transient elastography.
Fibrosis stages: F1 = mild fibrosis; F2 = moderate fibrosis; F3 = severe fibrosis; F4 = cirrhosis. ≥ denotes 'at least' the stated stage.
